# Short Chain Fatty Acids Effect on Chloride Channel ClC-2 as a Possible Mechanism for Lubiprostone Intestinal Action

**DOI:** 10.3390/cells9081781

**Published:** 2020-07-26

**Authors:** Marcelo A. Catalán, Francisca Julio-Kalajzić, María Isabel Niemeyer, Luis Pablo Cid, Francisco V. Sepúlveda

**Affiliations:** 1 Centro de Estudios Científicos (CECs), Avenida Arturo Prat 514, Valdivia 5110466, Chile; francisca.julio@gmail.com (F.J.-K.); miniemeyer@cecs.cl (M.I.N.); pcid@cecs.cl (L.P.C.); 2 Instituto de Fisiología, Facultad de Medicina, Universidad Austral de Chile, Valdivia 5090000, Chile

**Keywords:** ClC-2, intestinal electrolyte transport, short chain fatty acid, lubiprostone, butyrate, CFTR

## Abstract

Lubiprostone, a 20-carbon synthetic fatty acid used for the treatment of constipation, is thought to act through an action on Cl^−^ channel ClC-2. Short chain fatty acids (SCFAs) are produced and absorbed in the distal intestine. We explore whether SCFAs affect ClC-2, re-examine a possible direct effect of lubiprostone on ClC-2, and use mice deficient in ClC-2 to stringently address the hypothesis that the epithelial effect of lubiprostone targets this anion channel. Patch-clamp whole cell recordings of ClC-2 expressed in mammalian cells are used to assay SCFA and lubiprostone effects. Using chamber measurements of ion current in mice deficient in ClC-2 or CFTR channels served to analyze the target of lubiprostone in the distal intestinal epithelium. Intracellular SCFAs had a dual action on ClC-2, partially inhibiting conduction but, importantly, facilitating the voltage activation of ClC-2. Intra- or extracellular lubiprostone had no effect on ClC-2 currents. Lubiprostone elicited a secretory current across colonic epithelia that was increased in mice deficient in ClC-2, consistent with the channel’s proposed proabsorptive function, but absent from those deficient in CFTR. Whilst SCFAs might exert a physiological effect on ClC-2 as part of their known proabsorptive effect, ClC-2 plays no part in the lubiprostone intestinal effect that appears mediated by CFTR activation.

## 1. Introduction

Knowledge of how to manipulate pharmacologically intestinal transport processes is important for the rational design of drugs to combat the intestine’s disorders that can result in diarrheal disease or constipation. An example is cystic fibrosis (CF), an inherited disease caused by mutations that impair the function of CFTR, a Cl^−^ channel important in respiratory as well as intestinal fluid secretion. The search for drugs that target specific defects in the CFTR Cl^−^ channel protein has resulted in the discovery of modulators useful in the treatment of the disease [1,2]. Lubiprostone, a 20-carbon synthetic fatty acid with a keto group at position 15, was developed for the treatment of constipation [3]. Lubiprostone was proposed to act by promoting intestinal electrolyte and fluid secretion through the activation of Cl^−^ channel ClC-2 [4], but this mechanism has been disputed [5].

ClC-2 is one of four mammalian plasma membrane Cl^−^ channels belonging to the CLC family [6]. ClC-2 channels are present in the intestinal epithelium, where they have been proposed to play roles in fluid and electrolyte transport processes. A report suggesting that ClC-2 may provide an anion conductance pathway to mediate Cl^−^ secretion in the mouse small intestine was based on a purported apical location of the channel [7]. There is now ample evidence that ClC-2 is present at the basolateral membranes of epithelial cells in the jejunum and colon [8,9,10,11]. Expression of ClC-2 in epithelial cell lines also resulted in the basolateral membrane being driven by structural signals resulting in this subcellular destination quite generally [10,12]. The absence of ClC-2 from apical membranes in intestinal epithelia is inconsistent with a role in electrolyte secretion, as it is present in surface epithelial cells, rather than those from crypts [10], which are the site of fluid secretion.

Functional data implicating ClC-2 in an absorptive ion transport function in the colon, obtained using a *Clnc2^−/−^* mouse, also support a basolateral localization for this channel [11,13]. The ablation of ClC-2 in a different *Clcn2^−/−^* mouse model led to an increase in cAMP-dependent Cl^−^ secretion, and double mutants lacking ClC-2 and expressing the CF mutant CFTR-ΔF08 had a markedly decreased early lethality [14], presumably due to an alleviation of the intestinal obstruction that normally takes place in CF animals owing to decreased fluid secretion.

In view of the results described above, it is difficult to envisage that the activation of intestinal ClC-2 channels by lubiprostone could be the mechanism for its action as a drug to antagonize constipation, but the reality is quite the contrary. An alternative mechanism of action for lubiprostone has been proposed and supported by strong experimental evidence. In this scenario, lubiprostone would act as an agonist of EP_4_ receptors, increasing intracellular cAMPs promoting secretion through CFTR activation [15,16,17,18]. The strongest evidence for an action of lubiprostone through CFTR rather than ClC-2 activation comes from a study using mice that were genetically deficient in CFTR and biopsy intestinal tissue from CF patients [19]. Lubiprostone evoked a secretory response in mouse and human intestinal epithelia that was absent from CFTR null mouse tissue or that of CF patients carrying the CFTR∆F508 mutant. This work [19] also demonstrated that lubiprostone activated EP_4_ prostanoid receptor to induce an increase in cAMP.

All this evidence notwithstanding, the idea that lubiprostone might act directly through ClC-2 activation has persisted. Indeed, in a recent paper, Cuppoletti et al. [20] propose that lubiprostone acts at a fatty acid binding site in ClC-2, making its purported action on the channel direct and independent of cAMP and the EP_4_ receptor. The high expression of ClC-2 in the colon, a site of absorption of short chain fatty acids (SCFAs) from very high luminal concentrations, led us to explore the possible regulation of the channel by these compounds. In addition, we have probed the possible role of ClC-2 in the intestinal effect of lubiprostone, taking advantage of the availability of ClC-2 null mice. Our results show that ClC-2 can be both activated and blocked by SCFAs but not by lubiprostone. Our experiments using genetically modified mice show that the secretory effect of lubiprostone in the colonic epithelium is independent of the presence of ClC-2 but consistent with an activation of CFTR.

## 2. Materials and Methods

Heterologous expression and electrophysiology. HEK-293 cells were grown and transiently transfected with the ClC-2 cDNA for ClC-2∆77-86 from Cavia porcellus [21], GenBank sequence AF113529. The expression plasmid for constructs ClC-2 and πH3-CD8 to identify effectively transfected cells were as described previously [21]. The bath solution contained the following (mM): 140 NaCl, 2 CaCl_2_, 1 MgCl_2_, 22 sucrose, and 10 Hepes, pH 7.4 adjusted with Tris. The pipette solution (35 mM chloride) contained the following (mM): 100 sodium gluconate, 33 CsCl, 1 MgCl_2_, 2 EGTA, and 10 Hepes, pH 7.4 adjusted with Tris. Different concentrations of butyrate, acetate, propionate, and lactate were achieved by the equimolar replacement of sodium gluconate in the solutions. Changes in liquid junction potential were calculated [22] and corrected for when necessary.

Standard whole-cell patch-clamp recordings were performed as described elsewhere [23]. The voltage pulse generator and analysis programs were from Axon Instruments. The currents generated by transfection were not observed in non-transfected cells.

To extrapolate tail currents, double exponentials plus a constant term equation were fitted to the current deactivation time course and the tail current value calculated as the sum of the size of the exponential terms and the constant value. The tail current as a function of the voltage curve was analyzed by adjusting a Boltzmann distribution, as described [24].

Animals. All animal procedures were reviewed and approved by the local Institutional Animal Care and Use Committee (protocol 2016-01). The mouse facility at the Centro de Estudios Científicos (CECs) is AAALAC accredited and adheres to the guidelines in the Guide for the Care and Use of Laboratory Animals (the Guide, NRC 2011). Mice were bred at the Animal Facility of CECs from *Clcn2*^−/−^ [25] or *Cftr^tm1Eur^* [26] founders. Animals had access to food and water ad libitum.

Tissue isolation and Ussing chamber experiments. All procedures were carried out by trained personnel within approved premises. Mice (C57BL/6J background) were euthanized by cervical dislocation. The colon was excised and opened lengthwise through the mesenteric border; the mucosal surface of the distal colon was scraped with a glass microscope slide to obtain a partially stripped mucosal sheet. This type of preparation yields a largely mucosa-containing tissue suitable for the measurement of transepithelial electrical parameters [27].

The sheet was mounted on a tissue-holding slider (aperture 0.1 cm^2^) and inserted as a dividing membrane in a modified Ussing chamber (Physiologic Instruments Inc., San Diego, CA, USA). The bath solution bathing either side of the epithelial layer contained the following (in mM): 120 NaCl, 25 NaHCO_3_, 3.3 KH_2_PO_4_, 0.8 K_2_HPO_4_, 1.2 MgCl_2_, 1.2 CaCl_2_, and 10 D-glucose. Both hemichambers were continuously gassed with 5% CO_2_ and 95% O_2_.

A VCC MC2 amplifier (Physiologic Instruments Inc., San Diego, CA, USA) and Acquire & Analyse v. 2.3 software through a DI-720 data acquisition system (DataQ Instruments, Akron, OH, USA) were used to continuously measure the transepithelial potential difference (referred to the serosal compartment), clamp current at zero, and deliver 1 s, 10 μA pulses at 0.2 s intervals. The difference in current and voltage was used to calculate the tissue resistance and equivalent short-circuit currents according to Ohm’s law.

Apical 10 μM amiloride (Sigma-Aldrich, St Louis, MO, USA) was added to block Na^+^ current through the epithelial sodium channels (ENaC). Apical lubiprostone (Santa Cruz Biotechnology, Dallas, TX, USA) was used at 0.1 μM. Increase in intracellular cAMP was achieved with 1 μM forskolin (Merck, Darmstadt, Germany) and 100 μM isobutylmethylxanthine (IBMX, Sigma-Aldrich, St Louis, MO, USA). The KCNQ1/KCNE3 K^+^ channel was inhibited using 10 μM chromanol 293B (Tocris Bioscience, Bristol, UK). Carbachol 100 μM (Sigma-Aldrich, St Louis, MO, USA) was used to activate muscarinic cholinergic receptors.

## 3. Results

**Figure 1:**
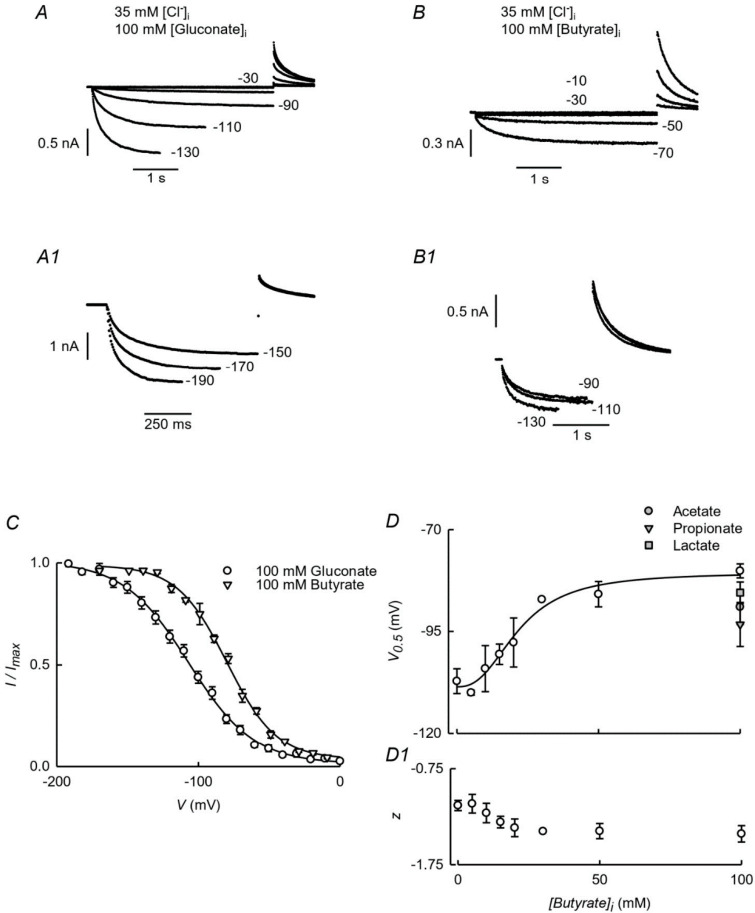
Effect of intracellular butyrate on ClC-2 currents. (**A**) and (**A1**): current traces mediated by ClC-2 elicited from a holding potential V_h_ of 0 mV in response to test pulses to the indicated potentials. Main activating pulses were followed by a pulse to 30 mV. The duration of the main activating pulses was increased at more positive voltages in order to approximate full activation of the conductance. The beginnings of the tail currents at 30 mV were moved to align them in the time axis. The intracellular solution contained 35 mM Cl^−^ and 100 mM gluconate. The bath solution contained 146 mM Cl^−^. Full details of the solution composition are given in the Methods section. (**B**) and (**B1**): as A and A1 but with 100 mM butyrate replacing gluconate in the intracellular solution. (**C**): Normalised tail currents, giving an estimation of apparent open probabilities, calculated as described in the text are given for experiments as those in A and B sections. The continuous lines are fits of Boltzmann distributions to the data (means ± SEM, *n* = 7 and 5 for experiments in gluconate and butyrate respectively). V_0.5_ (**D**) and slope factor (**D1**) parameters derived from the Boltzmann fits at various intracellular butyrate concentrations. Experiments obtained with 100 mM acetate, propionate and lactate in the pipette solution are also shown in **D**. Data are means ± SEM of 2–9 experiments.

High intracellular SCFA concentrations are likely attained in the transporting colonocytes during their absorptive process in the large intestine. Given the presence of ClC-2 in these cells [10], we have asked whether they might affect the function of the channel. Figure 1 shows current traces for HEK-293 cells expressing ClC-2 obtained by whole-cell patch-clamp recording with an intracellular solution containing 35 mM Cl^−^ and 100 mM gluconate (panels A and A1) or the same Cl^−^ concentration but 100 mM intracellular butyrate (panels B and B1). The values for the traces indicate the magnitude of the main activating voltage pulses, which are followed by steps to 30 mV, thus allowing the measurement of tail currents and therefore the degree of activation reached. Comparison of the traces obtained with intracellular butyrate and those with gluconate, that we presumed to be inert and impermeant, suggest that less hyperpolarization is needed to activate ClC-2 when the SCFA is present. This impression is confirmed by examining the tail currents at 30 mV as a function of the magnitude of the main activating pulse (Figure 1C). The activation curve for ClC-2 was displaced in the depolarizing direction when 100 mM butyrate was present intracellularly. The V_0.5_ shifted to a value of −80 ± 1.7 mV (*n* = 5) from −107 ± 3.3 mV (*n* = 7), the V_0.5_ measured when 100 mM gluconate was the main intracellular anion (means ± SEM, *n* is the number of experiments). Figure 1D shows that the butyrate effect follows a sigmoidal concentration dependence, with concentration giving a half-maximal effect EC_50_ at 22 mM and n_H_ value of 2.5. The valence derived from the slope factor is given in panel D1. Similar effects on V_0.5_ can be seen with other SCFAs, and those for 100 mM acetate, propionate, and lactate are also shown in Figure 1D.

**Figure 2:**
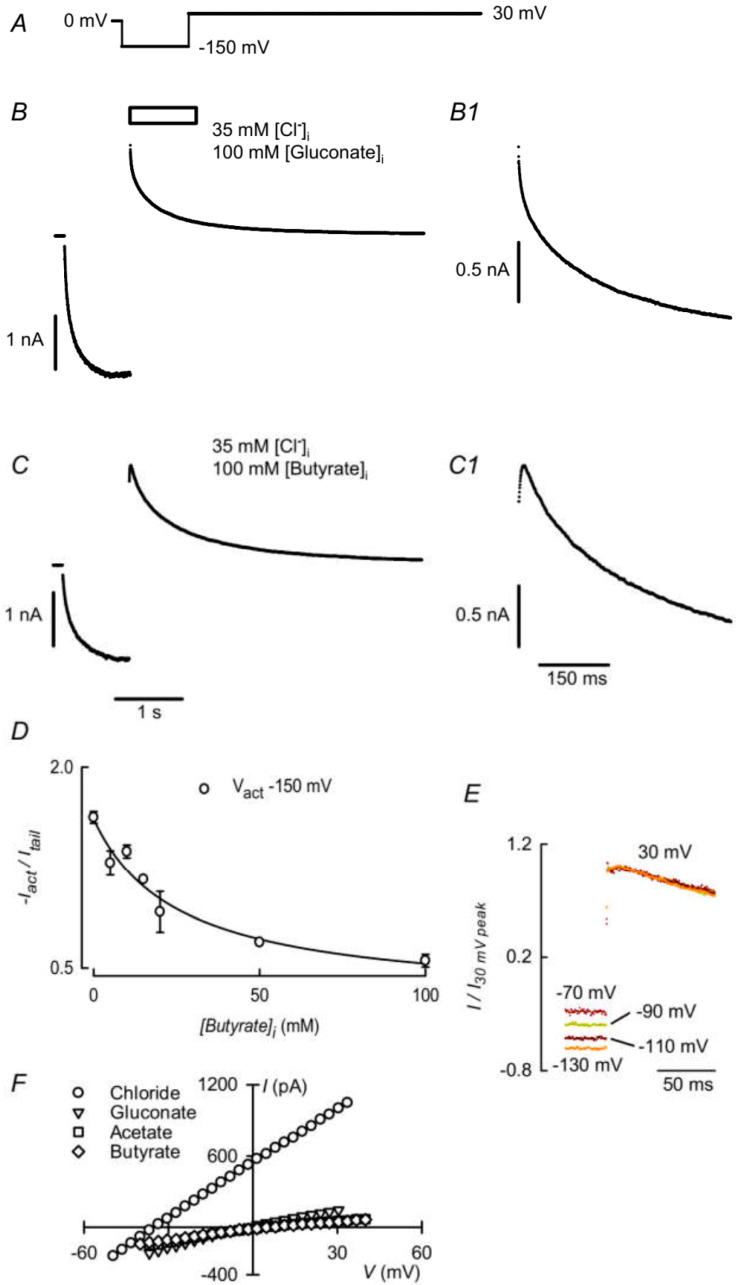
Inhibition of ClC-2 by intracellular butyrate is relieved at positive potentials. (**B**): current mediated by ClC-2 elicited by the voltage protocol shown in (**A**). The intracellular solution contained 35 mM Cl^−^ and 100 mM gluconate. The bath solution contained 146 mM Cl^−^. (**B1**): a magnified view of the time course interval indicated by the box above the trace in B is shown. (**C**) and (**C1**): as (**B**) and (**B1**) but with 100 mM butyrate replacing gluconate in the intracellular solution. (**D**). Ratio of the absolute value of the current elicited by a −150 mV activating pulses to those of the respective tail currents at various intracellular butyrate concentrations. Means ± SEM, *n* = 2–9. (**E**): traces of normalised tail currents in the presence of 100 mM butyrate after activation pulses ranging from −70 to −130 mV. (**F**): Effect of extracellular anion replacement on ClC-2 current vs. voltage curves. Stretches encompassing the reversal potential are shown for bath 146 mM Cl^−^ solution and after replacement of all but 16 mM Cl^−^ by gluconate, acetate or butyrate. The curves were obtained at the end 1.5 s, −130 mV activating pulse by means of a linear voltage ramp taking the potential to 30 mV in 25 ms.

Examination of the traces in Figure 1A,B shows that, in the presence of butyrate, the main activating pulses elicit smaller currents with respect to those of the tail currents at the depolarizing post-pulse than in the absence of the SCFA. This suggests that, in addition to the activating effect of the SCFA, butyrate might be blocking inward currents, i.e., anion efflux. That this might be the case is suggested by closer examination of tail currents in the absence or presence of butyrate (Figure 2B,C, respectively). The deactivation of the ClC-2 current after activation at −150 mV occurs monotonously during depolarization to 30 mV (initial portion expanded in Figure 2B1). With 100 mM intracellular butyrate, the tail current initially increases, as though recovering from the blockade, to then deactivate in the expected way (Figure 2C,C1). To quantify the apparent inhibition by butyrate, data comparing activation and deactivation were analyzed at several intracellular concentrations of butyrate, as shown in Figure 2D. The ratio of the absolute value of the activation current to that of the tail current decreases as intracellular butyrate concentration increases, consistent with the concept of butyrate acting as a blocker of ClC-2. The data can be described with a decreasing rectangular hyperbola with IC_50_ of 25 mM.

To gain an idea about the possible voltage dependence of butyrate’s blocking effect, we have examined the development of the tail currents at 30 mV after activating pulses of different voltages. Figure 2E shows traces of normalized tail currents after activation pulses ranging from −70 to −130 mV. It appears that the tail currents develop with similar kinetics after the different activating pulses, which would be consistent with low voltage dependence for butyrate inhibition. A similar result was obtained in four separate experiments.

Concerning SCFA permeability, butyrate and acetate are largely impermeant, as shown in panel F of Figure 2. The graph shows current vs. voltage curves recorded in a cell dialyzed with a pipette solution containing 35 mM Cl^−^ and 100 mM gluconate, with a bathing solution of 146 mM Cl^−^. The reversal potential of −37.7 ± 0.6 mV (mean and SEM of four separate experiments) is close to E_Cl_. Replacement of all but 16 mM Cl^−^ with the foreign anions butyrate or acetate displaced the reversal potential in the depolarized direction, consistent with P_X_/P_Cl_ ratios of 0.08 ± 0.03 (*n* = 4) and 0.07 ± 0.02 (*n* = 4) for butyrate and acetate, respectively, similar to that of the impermeant anion gluconate (0.08 ± 0.01, *n* = 4).

**Figure 3:**
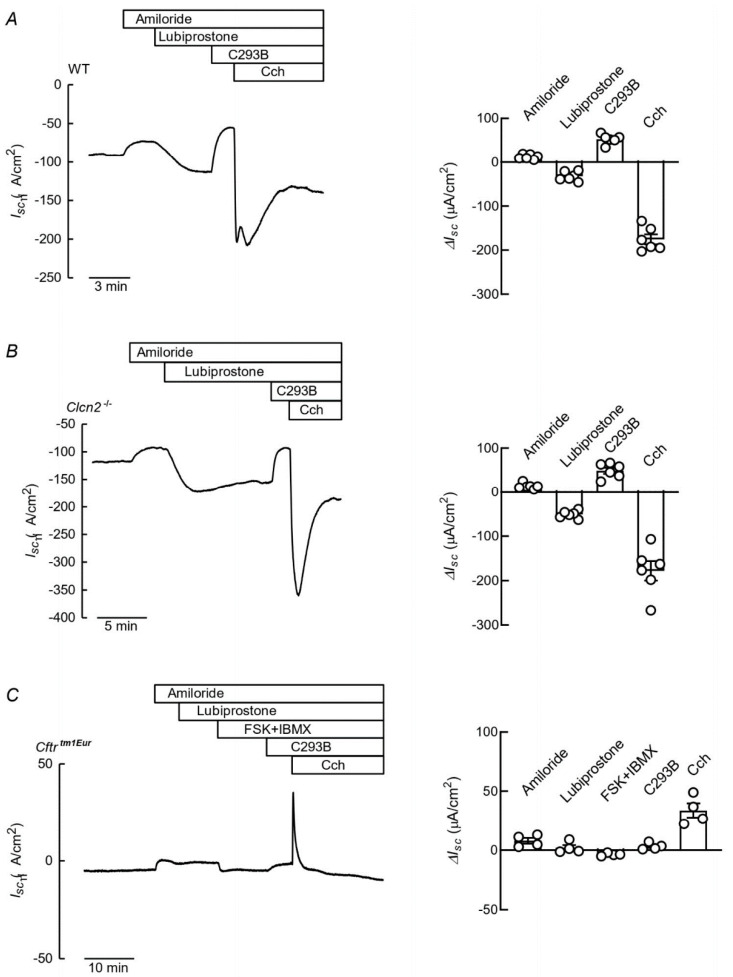
Effect of inactivation of Cl- channel ClC-2 on the effect of lubiprostone on anion secretion in the mouse colon. Traces showing recordings of short circuit currents (I_SC_) as function of time obtained using distal colon from WT (**A**) or *Clcn2*^−/−^ (**B**) mice mounted in Ussing chambers. Additions as shown in the upper bars, were 0.1 μM lubiprostone, 10 μM chromanol 293B (C293B) and 100 μM carbachol (Cch). All additions were made to the serosal side of the epithelium save for amiloride and lubiprostone, which was added apically. To the right of each trace corresponding bar graphs summarise the mean change in short circuit-current (∆I_SC_) after the different additions as means ± SEM of the six animals for each genotype. The only statistically significant difference was between the lubiprostone responses that were larger in tissues from *Clcn2*^−/−^ mice (P = 0.00355 by one-tailed *t*-test). (**C**): Effect of the same additions as in A and B tested using colon tissue isolated from *Cftr^tm1Eur^* mice that express the CFTR∆F08 mutant. Results summary on the right based on tissues from four animals.

Given that lubiprostone is a fatty acid, it is conceivable that a mechanism involved in its proposed role in the amelioration of constipation could be of that of a ClC-2 blockade, as seen here with SCFAs, and a consequent diminution in ion absorption. We have now used a *Clcn2* null mouse [25] to explore the role of ClC-2 in the intestinal effect of lubiprostone. Figure 3A shows an I_SC_ recording of the distal colon epithelium from a wild type (WT) mouse mounted in an Ussing chamber. After blocking ENaC-mediated Na^+^ absorptive current with 10 µM mucosal amiloride, addition of 0.1 µM lubiprostone elicited a negative current, consistent with anion secretion. This can be inhibited by the addition of 10 µM of chromanol 293B to block the KCNQ1/KCNE3 K^+^ channel. Further addition of carbachol (Cch, 100 µM carbachol) to the serosal solution to increase intracellular Ca^2+^ and activate K_Ca_3.1 resulted in an anion secretory current known to be mediated by CFTR [28], presumably activated by the previous addition of lubiprostone. The average change in short-circuit current (∆I_SC_) across the colon epithelium under the different conditions is given in the right-hand panel of Figure 3A. An experiment performed on colon tissue from a *Clcn2*^−/−^ mouse can be seen in Figure 3B. The same additions as in Figure 3A gave similar responses, revealing in particular that the anion secretory response to lubiprostone was still present in the epithelium lacking ClC-2. The panel to the right of the current trace gives the average changes in I_SC_. None of these differ significantly from those measured in the WT tissues, save the ∆I_SC_ in response to lubiprostone, that was significantly larger in the colon from *Clcn2*^−/−^ than in that from WT mice (*p* = 0.00355, by *t*-test). We also probed the effect of the drug in the colonic tissue of mice carrying the ∆F508 mutant of CFTR, the most common defect causing CF in humans. The lubiprostone effect was absent from the epithelia of these animals, in which increasing cAMP or addition of Cch also failed to evoke any anion secretory response, this last compound eliciting a K^+^ secretory current, as described previously [28].

**Figure 4:**
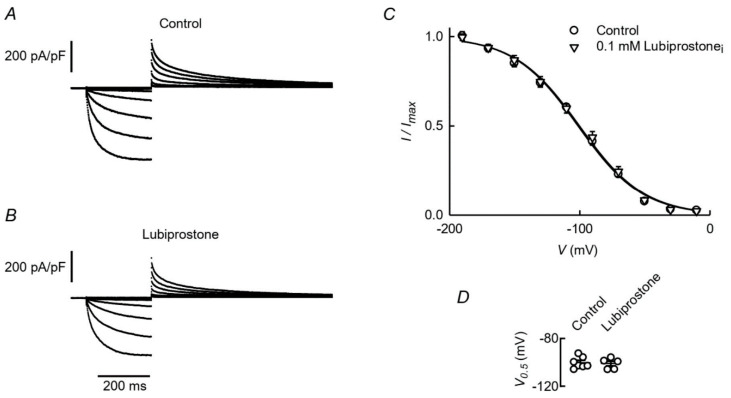
Effect of intracellular lubiprostone on ClC-2 mediated currents. Whole cell currents measured in HEK-293 cells transfected with ClC-2. (**A**,**B**): representative current traces, elicited from a V_h_ of −30 mV by test pulses ranging from −190 to −10mV in 20mV steps. These were followed by a pulse to 30mV. (**A**): control experiment with an extra- and intracellular solution containing respectively 146 and 35 mM Cl^−^. (**B**): a similar experiment except for the addition of 0.1 µM lubiprostone to the intracellular solution. (**C**): Tail currents plotted against the voltage of the main activating pulse are means ± SEM of 7 and 5 experiments, respectively. The lines are the averaged Boltzmann fits of the individual experiments whose V_0.5_ values are shown in (**D**).

Finally, we have tested for a direct effect of lubiprostone on currents mediated by ClC-2 when expressed in HEK-293 cells. A concentration of 0.1 µM lubiprostone was used that, independently of the interpretation of the mechanism of an eventual effect, has been shown to cause a near maximal effect [4,18,20]. Figure 4A,B show current traces from cells recorded under control conditions (A) or when 0.1 µM lubiprostone was present in the intracellular solution (B). The currents in both cases, activating slowly as voltages for the main pulse became more hyperpolarized, had the typical appearance of those mediated by ClC-2 channels and were not affected by intracellular lubiprostone. Sampling current after activation at −130 mV yielded an average of −2.08 ± 0.45 nA (*n* = 7) under control conditions and −2.10 ± 0.29 nA (*n* = 5) for cells with intracellular lubiprostone. In Figure 4C, the activation curves for the same set of cells are plotted and can be seen to be indistinguishable, with respective V_0.5_ values of −100 ± 1.8 and −101 ± 2.0 mV (Figure 4D).

**Figure 5:**
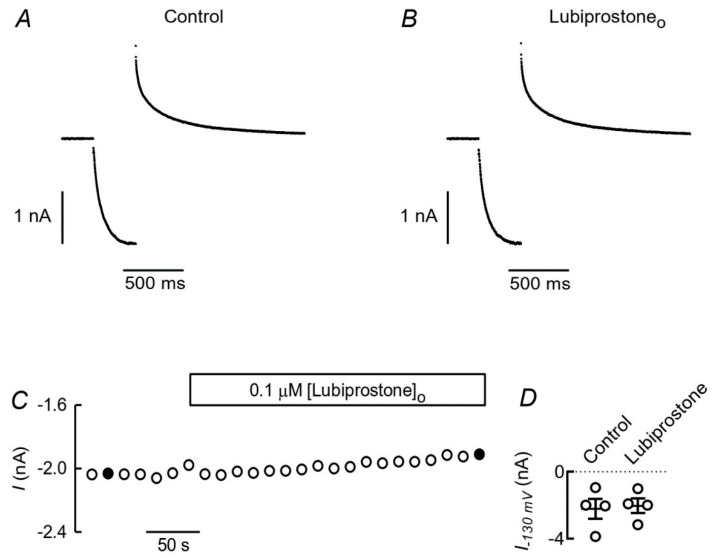
Effect of extracellular lubiprostone on ClC-2 currents. Whole cell currents measured in HEK-293 cells transfected with ClC-2. Traces in (**A**) and (**B**) were elicited from a V_h_ of −30 mV by test pulses to −130 followed by a step to 30mV and correspond to a control current and one taken after addition of 0.1 µM lubiprostone. Extra- and intracellular solutions were respectively 146 and 35 mM Cl^−^. They are part of a train of identical pulses given with a 15 s period. The complete experiment is reported in (**C**) through the steady state currents at the main activating pulse. In black the pulses illustrated in (**A**) and (**B**). (**D**): the effect of lubiprostone in separate individual experiments.

In Figure 5, on the other hand, the possible effect of external lubiprostone is tested. In A and B are current traces obtained by pulsing to -130 mV, followed by a deactivating pulse at 30 mV before (A) and after (B) superfusing a HEK-293 cell expressing ClC-2 with 0.1 µM lubiprostone. The currents in A and B are part of a train of pulses given at 15-s intervals. The measured currents for the complete series of pulses is given in C, where traces illustrated in detail in A and B are identified as filled circles. Similar results (Figure 5D) were obtained in four separate experiments that show that there was no significant effect of extracellular lubiprostone on ClC-2.

## 4. Discussion

The bacterial fermentation of undigested carbohydrates and proteins in the mammalian colon is responsible for the presence of short chain fatty acids (SCFA) in this segment of the intestine. Intraluminal concentrations of SCFAs in the colon can reach combined levels of the order of 150 mM, compared to <0.5 mM in the portal circulation [29,30]. More than 95% of the SCFA content of the distal gut is absorbed by epithelial transport involving both non-ionic diffusion and carrier-mediated transport [31]. The involvement of both Na^+^- and H^+^-coupled transporters has been proposed [32,33,34]. It is interesting that SCFA, although inhibiting Cl^−^ permeation through ClC-2, also behaves as an activator that enhances the sensitivity of the channel to voltage, such that it will become more active at less hyperpolarized potentials. ClC-2 has been proposed to act as the basolateral Cl^−^ efflux pathway in electroneutral NaCl absorption [11]. As SCFA stimulates electroneutral NaCl absorption in the colonic epithelium [35,36], our results showing that SCFAs activate ClC-2 channels agree with a pro-stimulatory effect of SCFA in NaCl transport in intestinal epithelia.

Although our results indicate that SCFAs regulate ClC-2 activity directly, an SCFA-dependent mechanism involving G-coupled receptors cannot be ruled out. Indeed, SCFAs not only act as energy sources [37] and histone deacetylase inhibitors [38] in enterocytes but also bind and stimulate G-coupled receptors [39]. GPR41, GPR43, and GPR109A are G-coupled receptors expressed in several cell types, including intestinal epithelial cells that are activated by SCFAs in the concentration range found in the intestinal lumen [40,41,42]. Little is known regarding the regulation of ClC-2 by G-protein coupled receptors, but it has been reported that the phosphorylation status of ClC-2 is regulated by different kinases such as PKA [43], JAK3 [44], SPAK and OSR1 [45], p34^cdc2^/cyclin B [46], and protein phosphatase 1 [46]. It is not known whether these additional putative effects targeting ClC-2 have any relevance for the physiological function of the channel.

It has also been proposed that lubiprostone promotes a functional switching from absorptive-to-secretory phenotype in jejunal villous and surface colonic epithelial cells by a mechanism that facilitates the internalization of key absorptive proteins such as NHE-3, DRA, and ClC-2, while stimulating the insertion at the plasma membrane of CFTR, NBCe1, and NKCC1, key proteins involved in intestinal solute secretion [47,48]. Lubiprostone effects would depend on a functional interplay between prostanoid receptors and cAMP-dependent protein kinase (PKA) [48], but it has been shown that, although ClC-2 is phosphorylated upon PKA activation in a heterologous system, there was no functional effect on the channel [43]. It must be pointed out that ClC-2 levels at the plasma membrane are dynamically regulated by specific endocytosis and exocytosis mechanisms, including the tyrosine endocytosis motif and intracellular ATP levels [49,50], so it is possible that an increase in intracellular cAMP induced by lubiprostone might result in the PKA-dependent endocytosis of native intestinal ClC-2 channels.

Concerning the mechanism of the direct effects of SCFAs on ClC-2, we have not investigated further the possible location of the site(s) of their action on the channel. The activation of ClC-2 is known to be dependent on voltage, intracellular Cl^−^, and extracellular H^+^. Activation by H^+^ has been linked to the neutralization of the so-called gating glutamate identified in ClC protein structures [51], which is conserved and functionally relevant in ClC-2 [52]. Intracellular Cl^−^ was proposed to favor this neutralization allosterically by interacting with the most intracellular of three putative anion binding sites of the channel’s selectivity filter (SF) [53]. More recent analysis from Arreola’s laboratory proposes that hyperpolarization drives intracellular Cl^−^ into the permeation pathway to displace the gating glutamate through an electrostatic effect, and this accounts for the voltage-dependent gating of ClC-2 [54,55]. The poor permeability of butyrate would suggest that the activating effect might be associated with an interaction with the permeation pathway at a site that does not sense the membrane potential, perhaps the innermost site of ClC-2 SF. Activation could then be interpreted as an allosteric effect, as propounded before for Cl^−^ [53], in which case relief of inhibition might be a flush-through effect of Cl^−^ influx rather than a direct voltage-dependent removal of the SCFA. An alternative interpretation, of activation taking place by partial voltage-dependent ingress of the SCFA into the pore without completely traversing it [55], however, cannot be discarded. Nevertheless, the similar degree of inhibition at various voltages evidenced by data in Figure 2E would militate against the concept of a voltage-dependent blockade and suggest a site that is superficial with respect to the electric field across the membrane.

Lubiprostone is a pharmacological agent used in the treatment of constipation [3] that has been proposed to act by promoting intestinal electrolyte and fluid secretion through the activation of Cl^−^ channel ClC-2 [4], a mechanism that is now disputed [5,56]. Indeed, ClC-2 channels are basolateral and required for the absorption of NaCl and KCl in the colon [11]. Consistent with a pro-absorptive role for ClC-2, cAMP-stimulated Cl^−^ secretion is increased in *Clcn2^−/−^* mice and early lethality of the *Cftr^tm1Eur^* mouse, that carries the CFTR-∆F08 CF mutant, is markedly ameliorated upon additional inactivation of *Clcn2* [14]. Lubiprostone effect has been studied in murine models of CF or in tissue from CF patients [19], which show that increased secretion promoted by the drug is dependent on, and caused by activation of, CFTR.

Despite the protracted dispute about the mechanism of lubiprostone action, it is surprising that its effect on intestinal ClC-2 had not been tested through the use of genetic inactivation in animals. Our results fill this gap and reveal that the presence of ClC-2 is not necessary for lubiprostone action in intestinal transport. Lubiprostone is capable of eliciting an anion secretory response in the mouse colon, and this effect is not only not curtailed in the absence of ClC-2 but enhanced. This is consistent with ClC-2 exerting a proabsorptive effect as it has been deduced from experiments with double mutants lacking both CFTR and ClC-2 [14] or directly in mice null for ClC-2 [11]. Our own control experiments using *Cftr^tm1Eur^* mice confirm a previous report [19] that any increase in intestinal anion secretion by lubiprostone requires the presence of a fully functional CFTR channel and is most probably due to an increase in cAMP, secondary to the activation of EP_4_-type prostanoid receptors.

The concept of lubiprostone as a specific activator of ClC-2 originates from work with T84 colonic carcinoma cells that express the channel but also on recombinant ClC-2 expressed in HEK-293 cells [4]. It has been pointed out [5] that reported results showed “linear, time-independent currents that differ markedly from ClC-2 currents” and, when tested on the typical well, characterized by ClC-2 slowly activating inwardly rectifying currents mediated by ClC-2 in heterologous expression in Xenopus oocytes, there was no effect of lubiprostone [18]. It could be argued that the discrepancy in results could originate from the different expression systems used. For this reason, we decided to revisit the problem of a possible direct effect of lubiprostone on ClC-2 heterologously expressed in HEK-293, be it by intra- or extracellular application of the compound. There was no effect of lubiprostone on ClC-2 activity in either of these experimental situations.

In conclusion, our results are inconsistent with an action of lubiprostone on the activity of the ClC-2 Cl^−^ channel as a possible explanation for a prosecretory intestinal effect. Clear effects of short chain fatty acids on the ClC-2 channel revealed here, on the other hand, might be of relevance to transport processes in the distal intestine. Our results using ClC-2 null mice point to a CFTR-mediated effect of lubiprostone, most likely mediated by prostanoid receptor activation [19], to underpin any prosecretory lubiprostone action.
